# Molybdate and Phosphate Cross-Linked Chitosan Films for Corrosion Protection of Hot-Dip Galvanized Steel

**DOI:** 10.1021/acsomega.3c01119

**Published:** 2023-05-24

**Authors:** Christian Fernández-Solis, Patrick Keil, Andreas Erbe

**Affiliations:** † Department of Interface Chemistry and Surface Engineering, Max-Planck-Institut für Eisenforschung GmbH, Max-Planck-Str. 1, 40237 Düsseldorf, Germany; ‡ BASF Coatings GmbH, Glasuritstraße 1, 48165 Münster, Germany; § Department of Materials Science and Engineering, NTNU, Norwegian University of Science and Technology, 7491 Trondheim, Norway

## Abstract

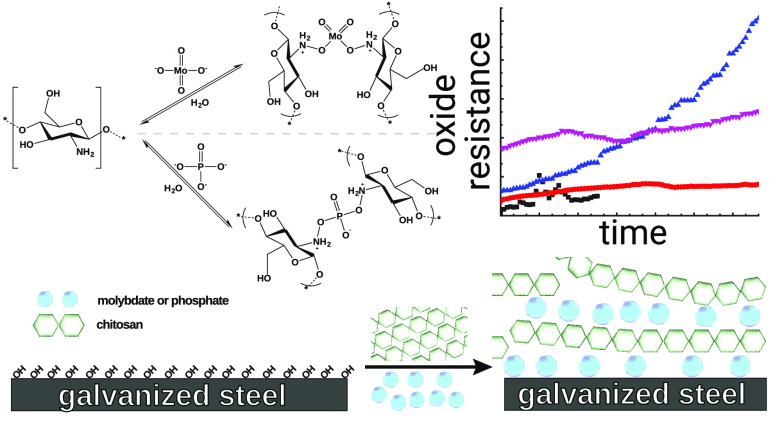

Environmentally friendly and sustainable methods to protect hot-dip galvanized (HDG) steel from corrosion are extensively studied. Films of the biopolymer polyelectrolyte chitosan were ionically cross-linked in this work with the well-known corrosion inhibitors phosphate and molybdate. Layers on this basis are presented as components in a protective system and could, e.g., be applied in pretreatments similar to a conversion coating. For the preparation of the chitosan-based films, a procedure involving sol–gel chemistry and wet-wet application was utilized. Homogeneous films of few micrometers thickness were obtained on HDG steel substrates after thermal curing. Properties of chitosan-molybdate and chitosan-phosphate films were compared with purely passive epoxysilane-cross-linked chitosan, and pure chitosan. Delamination behavior of a poly(vinyl butyral) (PVB) weak model top coating studied by scanning Kelvin probe (SKP) showed an almost linear time dependence over >10 h on all systems. Delamination rates were 0.28 mm h^–1^ (chitosan-molybdate) and 0.19 mm h^–1^ (chitosan-phosphate), ca. 5% of a non-cross-linked chitosan reference and slightly higher than of the epoxsyilane cross-linked chitosan. Immersion of the treated zinc samples over 40 h in 5% NaCl solution yielded a 5-fold increase of the resistance in the chitosan-molybdate system, as evidenced by electrochemical impedance spectroscopy (EIS). Ion exchange of electrolyte anions with molybdate and phosphate triggers corrosion inhibition, presumably by reaction with the HDG surface as well described in the literature for these inhibitors. Thus, such surface treatments have potential for application, e.g., in temporary corrosion protection.

## 1 Introduction

Hot-dip galvanized (HDG) steel is the most widely used alloy with worldwide presence and many technological applications. However, it is susceptible to corrosive processes under normal environmental conditions, and for this reason, great efforts have been advocated to inhibit or minimize these degrading effects. Important recent developments, triggered in part by changes in societal demand and global legislation, drive development of more environmentally friendly and sustainable coating systems with a broad raw materials base.

Conversion coating of HDG steel is usually performed by phosphating.^1−4^ Molybdate is a well-known corrosion inhibitor^5−7^ and arises as an alternative to traditionally used chromates which are phased out^8^ and as a complementary option to phosphate-based passivation methods. In the past decades, molybdates have been exhaustively studied and have successfully been used as corrosion inhibiting components, e.g., in antifreeze solutions in automobile cooling systems, and in aluminum alloys to increase pitting potential and reduce passive current.^9−16^ Molybdate has been used as an additive in phosphate conversion to accelerate the phosphating process,^17,18^ and it has been reported to enhance corrosion resistance of zinc and several other metals.^10,11,19,20^ Furthermore, molybdates have been used for protection of several alloys in different systems as less aggressive components.^10−12,14,15,21^ For improving corrosion protection, phosphates have been used, e.g., in the cross-linking of lignin.^22^ Phosphate-containing polymers are other promising corrosion inhibitors.^23^

Chitosan is a soluble derivative of a highly available natural polysaccharide: chitin.^24−30^ It has been extensively investigated because of its film-forming properties, its adhesion to surfaces, its antimicrobial effects, its biodegradability, its absence of toxic effects, and the ease of chemical modification of its functional groups.^31−34^ Employment of chitosan-based films for the protection of aluminum alloys has been investigated,^35^ and chitosan was used as an additive in epoxy coatings.^36^ A chitosan-silane hybrid film for corrosion protection of zinc with favorable electrochemical properties and low cathodic delamination rates was reported,^37^ as it may dampen extreme pH values during delamination.^38^ The incorporation of organic corrosion inhibitors into chitosan decreased corrosion rates of zinc.^39,40^ Similar effects were found for cyclic oligosaccarides;^41,42^ in this case, corrosion inhibition has been attributed to a decreased amount of point defects in the native oxide layer.^43^ Chitosan and other chitin-based compounds are also known for their high affinity to heavy metals, e.g., zinc, chromium, gold, and molybdenum, which has led to a wide range applications for environmental remediation.^19,44^ Chitosan with phosphate has been used, e.g., to tailor corrosion of magnesium alloys,^45−48^ titanium alloys,^49−51^ or stainless steels,^52−54^ for implants. Phosphorylated chitosan films,^55,56^ chitosan films with conductive polymers,^57^ or chitosan with organic corrosion inhibitors^58^ have also been used in corrosion protection.

Several applications for chitosan-based systems containing molybdate, phosphate, or both have been patented such as treatment of hyperphosphatemia based on phosphate-binding chitosan,^59^ aqueous treatment solution of chitosan and phosphonic acids for corrosion protection of aluminum and aluminum alloys,^60^ and chitosan systems containing molybdate to prevent scale formation in squeeze treatments for oil production and in industrial water treatment.^61^

**Figure 1:**
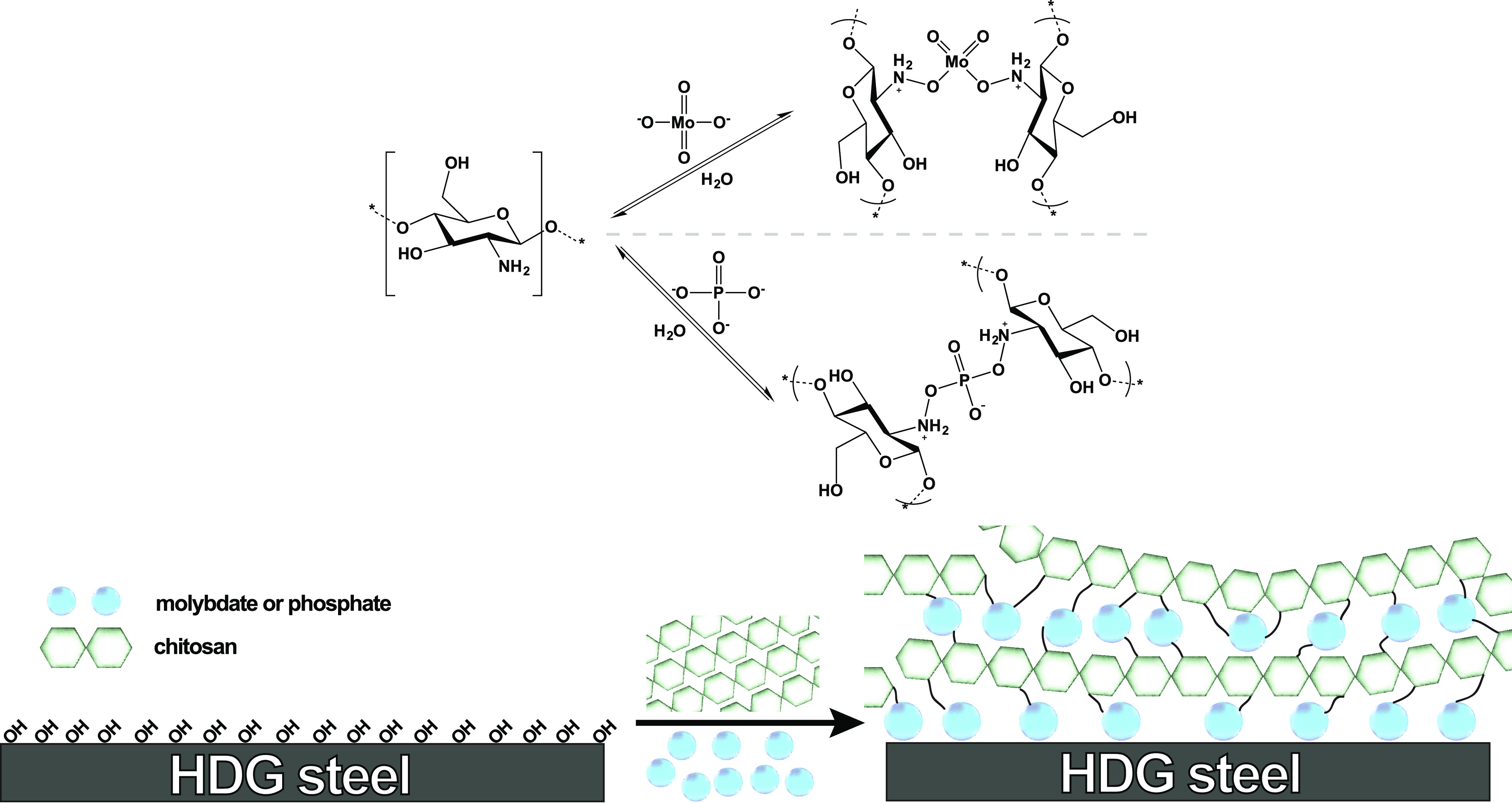
Schematic representation of the incorporation of phosphate and molybdate anions within chitosan deposited on a HDG steel substrate.

In this work, chitosan-based films were deposited on a HDG steel sample via a wet-wet application process. Cross-linking was enhanced by thermal curing. Figure 1 shows a representation of the incorporation of phosphate and molybdate into chitosan^62^ using ionic cross-linking. Ionic cross-linking is a standard method of cross-linking, e.g., in hydrogels.^63−65^ Ionic cross-linking is particularly well established for the chitosan-phosphate system^66−68^ but is also reported for chitosan-molybdate.^69^ Previous applications have however not focused on corrosion protection. The cross-linking of chitosan by an epoxysilane was investigated in detail elsewhere;^37^ this system is used here also for comparison. As opposed to the chitosan-phosphate and chitosan-molybdate systems, chitosan-silane is not supposed to release corrosion inhibiting compounds during corrosion processes.

A main aim of this work is to broaden the data basis for understanding the chemical processes during cathodic delamination by comparing systems with well-defined interfacial linkage,^70−73^ thus broadening the understanding of the chemical processes during cathodic delamination in the presence of weak model top coatings.^74^ Deadhesion kinetics with a weak model top coating was characterized by scanning Kelvin probe (SKP) experiments, in line with community standards.^75^ SKP data are complemented with a characterization of the film’s electrochemical behavior in a corrosive environment by electrochemical impedance spectroscopy (EIS). The development of a strongly protective, commercially competitive system was not a goal of this study, and neither was a full structural characterization of the resulting coatings. Application-wise, such physically cross-linked systems can more easily be removed as chemically cross-linked systems and are potentially suitable for application in temporary corrosion protection where coatings are used over time scales of weeks to months.

## 2 Results and Discussion

### 2.1 Preparation

Preparation of the investigated films is based on a 10 g/L chitosan solution in 0.2 M acetic acid. This solution was mixed 1:1 (v/v) with 0.5 M solutions of sodium phosphate or sodium molybdate, respectively, to yield a ratio of 0.05 mol inhibitor (phosphate or molybdate) per g of chitosan. This ratio corresponds to a molar ratio of ∼10:1 inhibitor:glucoside monomer unit. For preparation of coatings, ∼3 mL of the respective inhibitor-chitosan solution was spread over an HDG sample, left in contact for few minutes, and removed by a spiral bar coater of 10 or 12 μm. Films were left to dry at room temperature for 15 min and then thermally cured for 2 h at 100 °C, finally yielding films of ∼3 μm thickness. Full details of the preparation are given in section 4. No structural characterization of the prepared films was conduced in this work due to the complexity of the task.

**Figure 2:**
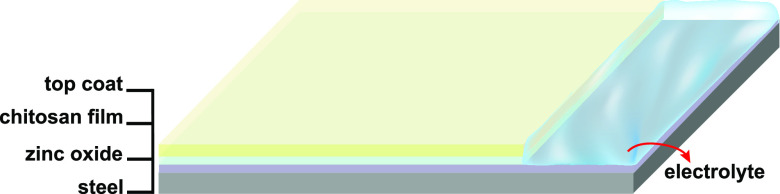
Representation of the layer composition of the sample used for delamination studies by SKP measurements. At the defect site, a solution of 5% NaCl was added to initiate cathodic delamination.

For delamination studies, the chitosan based films were further coated with a ∼20 μm thick top coat of poly(vinyl butyral) (PVB) from alcoholic solution. An artificial defect was prepared in these PVB layers. PVB as a weak model top coat avoids spreading of the electrolyte. Delamination rates on PVB are, however, supposed to be orders of magnitude higher than on strong, highly cross-linked polymer coatings with strong barrier properties and good adhesion.^75^Figure 2 shows the basic setup of the samples for delamination studies.

### 2.2 Cathodic Delamination Kinetics

**Figure 3:**
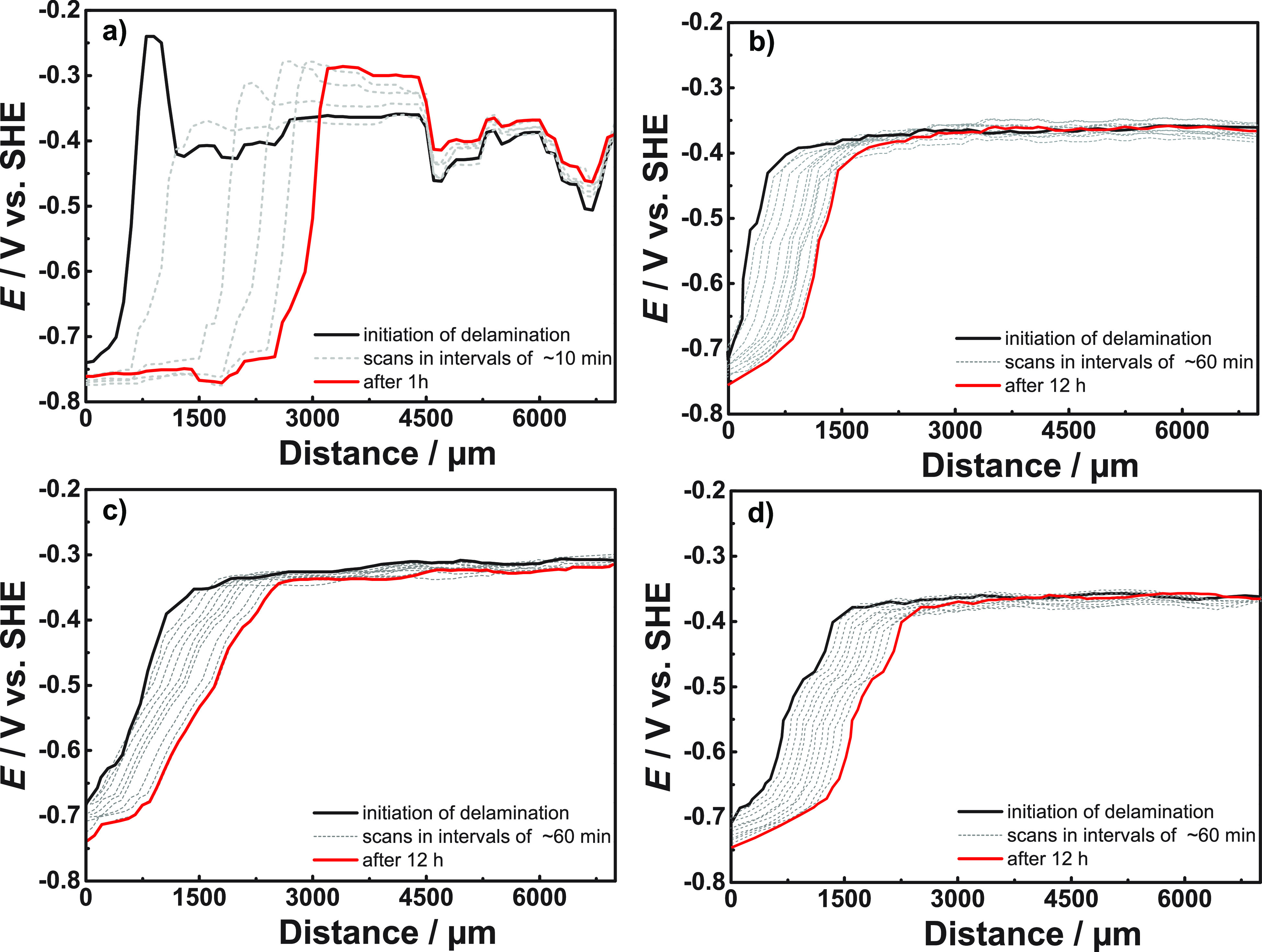
SKP delamination profiles of (a) unmodified chitosan film used as a reference, (b) chitosan-silane, (c) chitosan-molybdate, and (d) chitosan-phosphate on HDG, all samples top-coated with PVB and at relative humidity of ∼95%. The defect is in contact with 5% NaCl. “Initiation of delamination” is defined here as the first measurement for which a shift in the curve was detected.

The stability of the chitosan-based films was assessed by performing cathodic delamination experiments using SKP. Figure 3a shows the SKP potential profile during propagation of a delamination front from an artificial defect filled with 5% NaCl for a sample coated with unmodified chitosan, used as a reference. Figure 3b–d show the SKP potential profiles of chitosan-silane, chitosan-molybdate, and chitosan-phosphate coated samples, respectively. The profiles were obtained by scanning along the surface from the generated defect as explained elsewhere.^37,75^ The recorded potentials were plotted as a function of distance from the defect for different intervals of time. The first measurement included is the first measurement for which a shift in the potential profile was detected. The time between start of the experiment and initiation of delamination has not been investigated systematically in this study. During the first hours of the experiment, the potential recorded for all chitosan-based films is ∼−0.35 V vs SHE, which corresponds to the potential of an intact zinc interface on galvanized steel, with absence of active corrosion.^76^ Because of the high humidity in the chamber and the presence of electrolyte at the defect, at some point, cathodic delamination is initiated. Thus, the potential corresponding to freely corroding zinc is observed (∼−0.7 V vs SHE) and a propagation front is recorded by monitoring the propagation of the inflection potential between the section of the intact and delaminated portion of the film. Chitosan films containing epoxysilane, molybdate, and phosphate exhibit ∼50 mV less negative potentials than the reference near the defect, and a quite homogeneous negative potential at the side of the intact polymer. Both potentials result from a combination of kinetic parameters.^75,77^ Overall, the difference of potential between the active site of the substrate (defect) and the site of intact polymer (metal–polymer interface) is reduced, which is reflected in lower delamination rates, most likely because of inhibition of oxygen reduction.^78^

**Figure 4:**
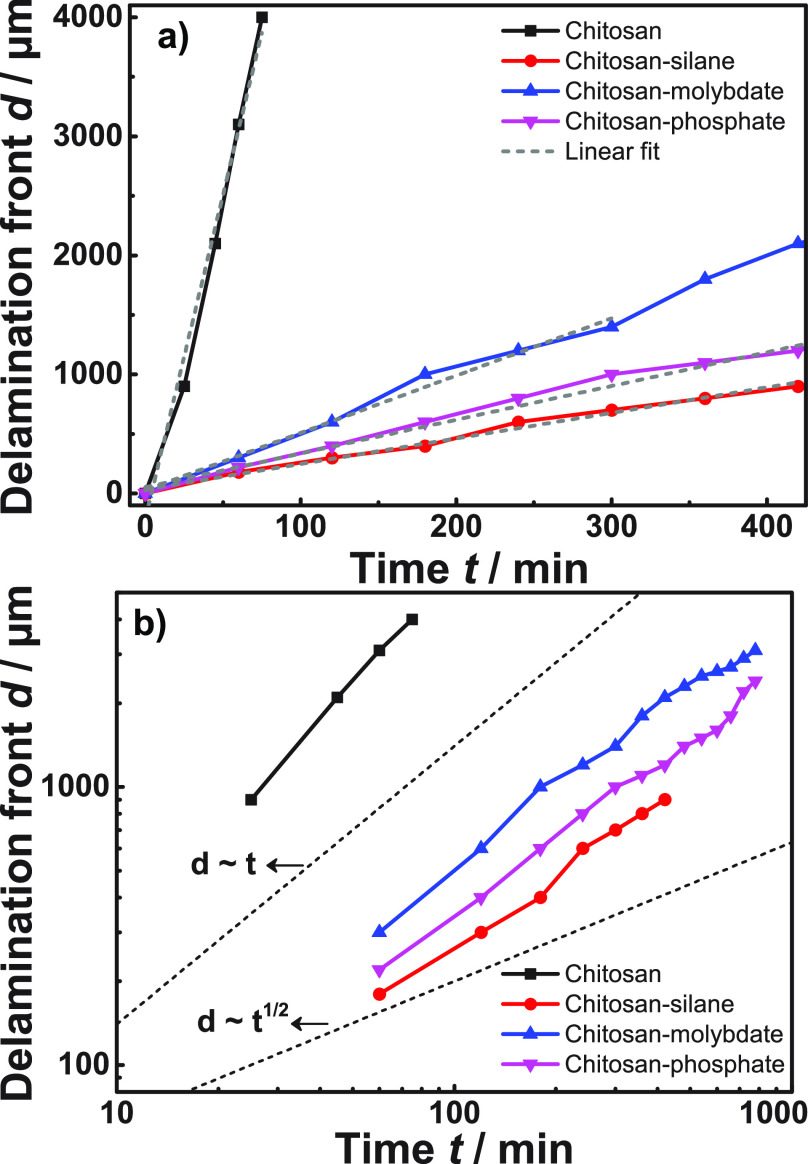
Progress of the delamination front for different samples (a) on linear scale and (b) on a double logarithmic scale. In plot (b), dotted lines show the expected slopes for *d* ∝ *t* and *d* ∝ *t*^1/2^, as indicated in the graph. A PVB top coat was present on all samples, see section 4.

The progress of the delamination front with time *t* for each film is plotted in Figure 4a. For all systems, the initial delamination rate *r* was determined from the slope of a linear fit such as in Figure 4a; results are summarized in Table 1. In order to determine the mode of delamination, Figure 4a is replotted with a double logarithmic scale in Figure 4b.^37,79^ The slopes determined from linear fits of Figure 4b provide the exponent α of the dependence of delamination front position *d* on time *t* of the delamination experiment, *d* ∝ *t*^α^; parameters are tabulated in Table 1. All chitosan-based films follow a delamination close to *d* ∝ *t*^1^ (α ≈ 1), which is traditionally associated either with a first-order reaction as rate-determining, or short-distance ionic migration.^37,79^ Therefore, a diffusion controlled process, which should progress as *d* ∝ *t*^1/2^ (α = 1/2),^37,79,80^ cannot be considered the rate determining mechanism. A very recent suggestion considers incorporation of cations into the metal oxide as rate determining for cathodic delamination.^81^ For chitosan films cross-linked with molybdate and phosphate, rate determining could be short-distance ionic migration that occurs as result of the anion’s release from the chitosan matrix and a subsequent reaction with the HDG surface as well described in the literature on molybdate as corrosion inhibitor.^5−7^ However, for the case of chitosan-silane hybrid, this short-distance migration is probably accompanied by a first-order reaction as a result of the breakage of siloxane bonds.^37,74^ So far, it is difficult to see the difference of cation insertion in line with ref (81) as a direct result of the varying of the anion as done here. This study can therefore neither support nor contradict the hypothesis of cation insertion as the rate-limiting step.^81^

**Table 1:** Initial Delamination Rates *r*, Exponent α of Time Dependence and Their Standard Deviations for Different Samples^a^

sample	*r*/mm h^–1^	α
chitosan	3.3 ± 0.2	0.96 ± 0.08
chitosan-silane	0.13 ± 0.01	0.85 ± 0.05
chitosan-molybdate	0.28 ± 0.01	0.82 ± 0.07
chitosan-phosphate	0.19 ± 0.01	0.94 ± 0.06

a All samples were top coated with PVB, see section 4.

The initial delamination rate of samples covered with cross-linked chitosan-based films decreased to <10% of the rate of the bare chitosan reference. The delamination rate found here for the reference system is comparable to the rate reported in previous works.^37^ The lowest delamination rate with a value of (0.13 ± 0.01) mm h^–1^ was obtained for the purely passive reference chitosan system that was epoxysilane cross-linked. The delamination rate of the chitosan-phosphate system is (0.19 ± 0.01) mm h^–1^ slightly higher, and the rate of the chitosan-molybdate system is (0.28 ± 0.01) mm h^–1^, circa twice as high as the lowest rate observed. Generally, delamination rate decreases as cross-linking density increases.^70,74,82^ Despite the fact that diffusive processes are not rate-determining, it is very probable that slow processes are involved such as diffusion of the ions formed in the first hours of the delamination and oxygen transport. At this point, deposition of phosphate and molybdate at the metal–polymer interface cannot be discarded.

### 2.3 Time-Dependent Barrier Properties Evaluated by EIS

**Figure 5:**
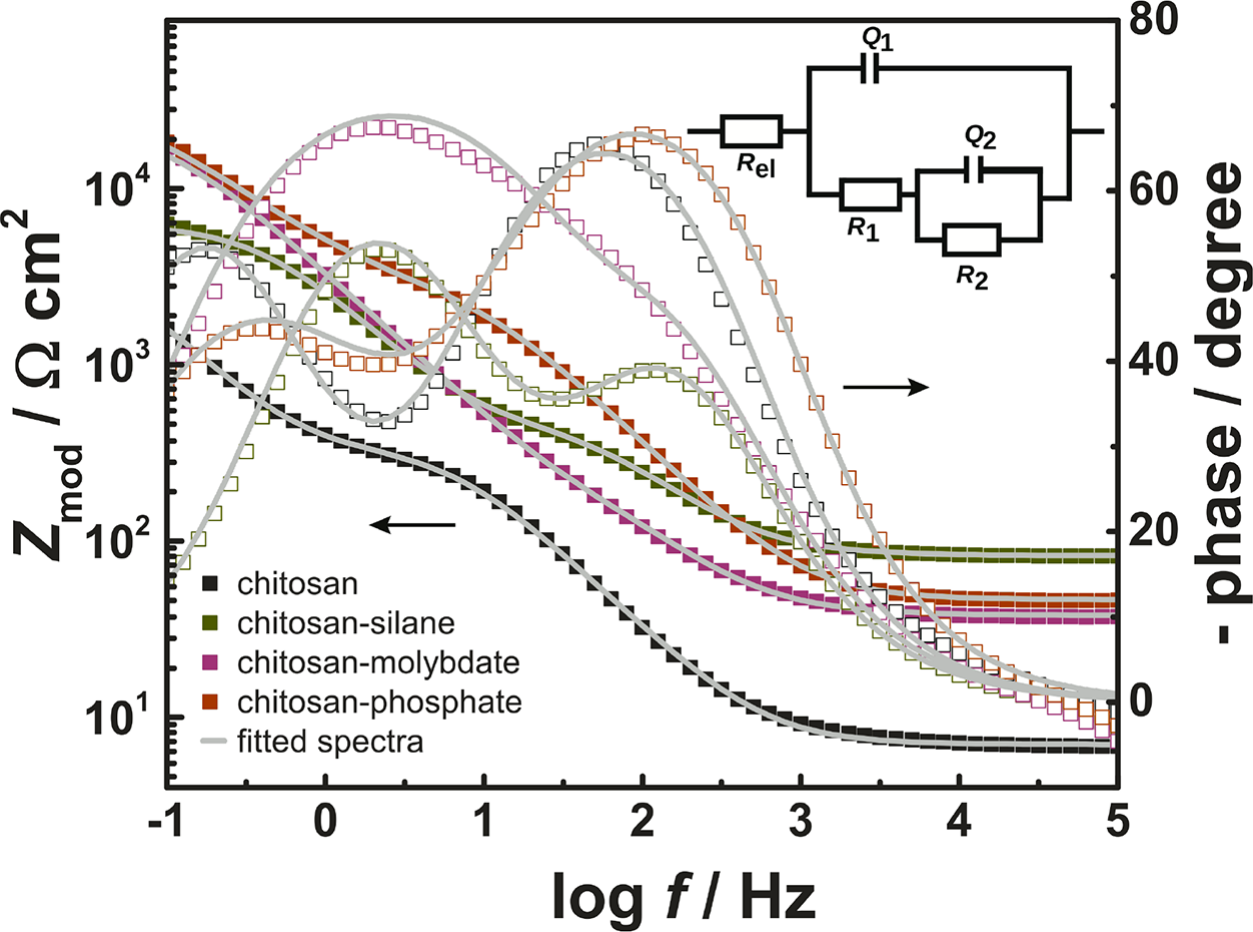
Bode plots after 30 min of immersion in 5% NaCl of a sample coated with unmodified chitosan. Inset: Equivalent circuit used to fit EIS spectra, *R*_el_ – electrolyte resistance, *R*_1,2_ – resistances of different components, *Q*_1,2_ – constant phase elements of different components.

Figure 5 shows the impedance spectra for the films after 30 min of immersion in 5% NaCl. As inset, the equivalent circuit used in this work to fit all time-dependent EIS measurements is displayed. The system contains obviously two RC circuits, as it contains two time constants. With its complex composition, the interpretation of these elements is not straightforward, which is why they are labeled 1 and 2. The polyelectrolyte layer can take up water and depending on the swelling, the system may behave in a similar way as a polymer brush, where a concentration gradient of monomers as a function of distance is expected.^83−85^ Furthermore, during heat treatment during preparation, oxide may grow from the metal into the polymer film. The two elements must therefore not necessarily be directly related to one pure layer. Also, the charge transfer resistance should be contributing to the impedance. The system under investigation here is notably different from the usual systems used in commercial applications, as it consists of a comparably thin organic coating, which can easily take up water. This system may be a system with limits in interpretation of the classical equivalent circuit elements. Solvated chitosan itself shows complex dynamics at frequencies below 1 MHz, with several relaxation modes leading to frequency-dependent changes in dielectric properties and hence capacitance.^86−89^ Since the dynamics change during drying,^90^ it is anticipated that they also changes during hydration. Relaxation of a dielectric process can be incorporated into equivalent circuit models by considering explicitly a frequency-dependent capacitance,^91^ which was not done here because of the large number of parameters required in the fitting.

From the viewpoint of corrosion protection, the equivalent circuit element with the highest resistance is important, as it will in long-term experiments contribute most toward keeping dissolution currents low. In this work, no reference of bare HDG was included. Comparison of the polarization resistance for pure zinc, chitosan-coated zinc, and siloxane-cross-linked chitosan coated zinc shows the expected decrease of the corrosion current density with the latter coating by more than 1 order of magnitude.^37^

A first feature worth noting is the differences in the high frequency impedance of the different systems. As this region is usually associated with the solution resistance, and similar cells and setups have been used here, one expects the solution resistance to agree and not to differ by ca. 1 order of magnitude. A similar difference has been observed before for the chitosan and chitosan-silane systems^37^ and has been attributed to the effect of the different water uptake has on the polyelectrolyte behavior. Alternatively, the differences may be related to a relaxation process in the at least partially hydrated polymers at higher frequencies. Relaxation modes in solvated chitosan at frequencies on the order of 10^8^–10^9^ Hz have been observed,^92,93^ i.e., at frequencies much higher than the frequencies used in this work. These modes would thus still be expected to contribute to an increased impedance above the solution resistance at the highest frequencies used in this work, and could be different in the different systems. Because of the observed differences, we use here the term electrolyte resistance for the limit of the impedance at the highest frequencies used here.

**Figure 6:**
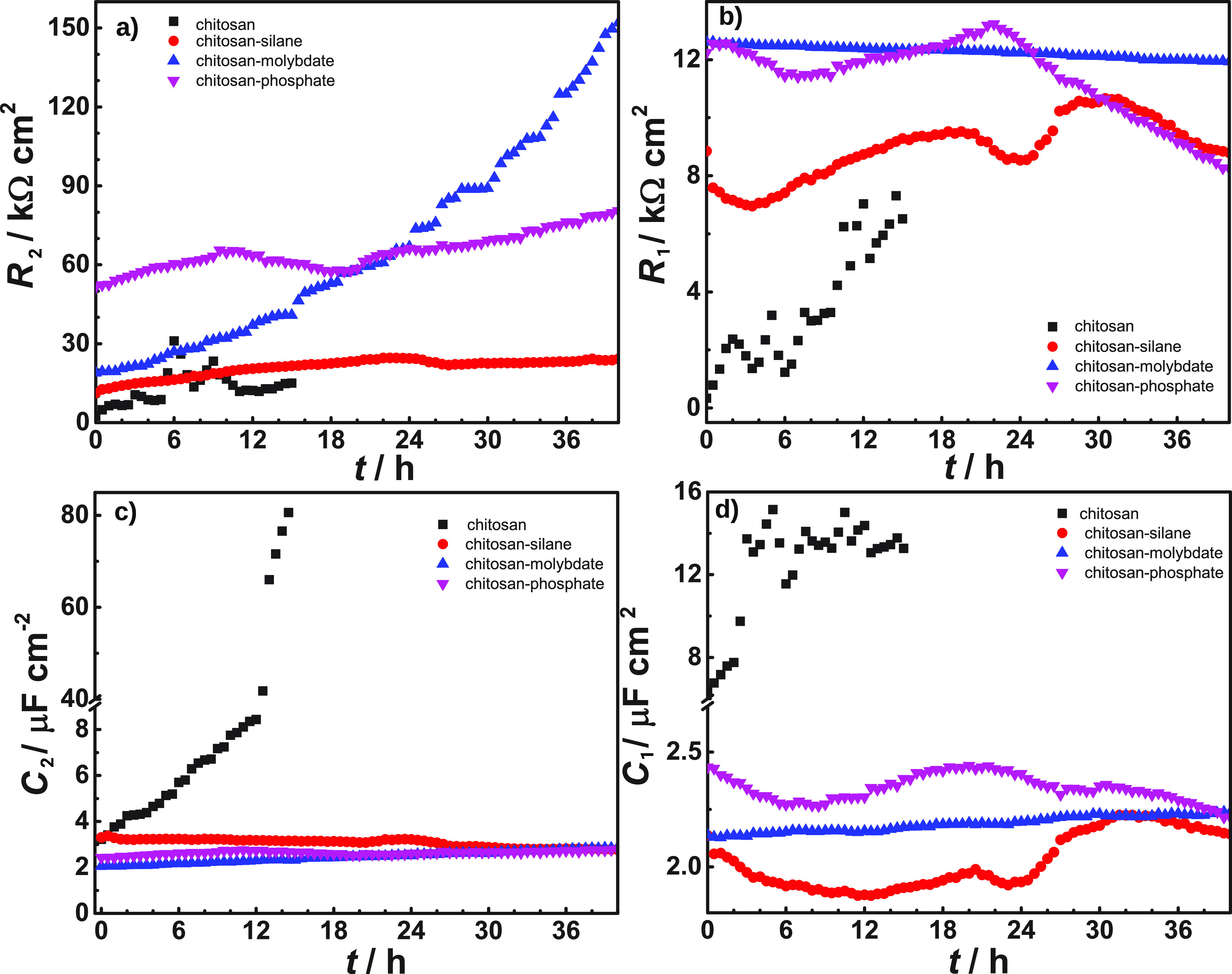
Time dependence of EIS fitting parameters obtained using the equivalent circuit in Figure 5; (a) *R*_2_, (b) *R*_1_, (c) *C*_2_, and (d) *C*_1_, all after exposure to 5% NaCl monitored over 40 h.

Figure 6a and b show the area-normalized resistance values for each sample. Figure 6c and d show the area-normalized capacitances. The constant phase elements, *Q*_1_ and *Q*_2_, were converted to area normalized capacitances *C* using the equation , where *A* is the sample area, *R* the respective parallel resistance, and *n* the exponent of the constant phase element; while more sophisticated interpretations of the constant phase element capacitance exist, see, e.g., discussion in ref (94), this simple conversion is judged sufficient for discussing trends throughout exposure. The exponents *n* of the constant phase elements of *Q*_2_ were 0.88...0.96 (chitosan), 0.9...0.94 (chitosan-silane), 0.8...1 (chitosan-molybdate; spectra with individual outliers down to 0 were discarded), and 0.75...0.95 (chitosan-phosphate); *n* for *Q*_1_ were typically lower, 0.81...0.88 (chitosan), 0.75...0.79 (chitosan-silane), 0.73...0.82 (chitosan-molybdate), and 0.8...0.83 (chitosan-phosphate). Lower values correspond to a wider distribution of time constants, implying that the underlying structural elements leading to *Q*_1_ have a stronger disorder. Strong disorder is a typical feature of solvated polymer chains, so it is likely that these contribute to *Q*_1_. Exponents showed, only in few cases, larger changes in the initial 3 h of the experiments, but these changes are not reflected in the calculated capacitances, which shall mainly be considered for further discussion. An exponent *n* as low as 0.75 already indicates significant deviation from capacitive behavior; nevertheless, trends in the curves can still be extracted and interpreted in relation to changes in the system.

The most remarkable feature in the time series is a constantly increasing *R*_2_ for chitosan-molybdate films. Moreover, *R*_1_ stays high during the whole experiment, with a ∼5% decrease. Both capacitances of chitosan-molybdate increase during the experiment (*C*_2_ by ∼50% from ∼2 to ∼3 μF/cm^2^, *C*_1_ by ∼5%). Increased capacitance with decreased resistance is expected for the uptake of water by the polymer, or a restructuring of the polymer; the trends are consistent with a relation of *R*_1_ and *C*_1_ to the polymer, though it is not given that only the polymer contributes. Increasing capacitance with at the same time increasing resistance can be a sign of a chemical transformation, e.g., the formation of passive molybdate products at the metal–polymer interface. Formation of passive products here must be a result of the release of molybdate ions from the chitosan matrix, especially when considering the other systems in comparison. The fact that an increase in resistance occurs over a prolonged period is related to the large capacity of the chitosan for molybdate storage. After long immersions, the passive layer formed by the molybdate obtains a certain stability. Detailed studies of the electrochemical mechanisms of corrosion inhibition by molybdate for related systems are available in the literature^6,7^ and not subject of this study.

The chitosan-phosphate film shows nonmonotonous trends in all quantities. *R*_2_ as the largest resistance increases by ∼50% from its initial value, after an intermediate decrease. The initial changes could be associated with release of phosphate from the chitosan matrix. These phosphates can form passive products at the metal–polymer interface, similar to that of conversion coatings. Such conversion could lead to the low delamination rate that chitosan-phosphate films exhibit in the SKP experiments. The continually decreasing capacitance observed in Figure 6d indicates that phosphate products are still being formed even after 24 h.

In chitosan-silane coated samples, the difference between the two resistances *R*_2_ and *R*_1_ is much lower than for chitosan-molybdate and chitosan-phosphate. All quantities show fluctuations without strong trends. Nonuniform formation of corrosion products may contribute to these trends.

The unmodified chitosan film exhibits an increase of both capacitance and both resistance values, which starts to scatter right after the first hours of exposure to the electrolyte, indicating dissolution of the chitosan layer. The increase of both resistance values may be related to the formation of oxides after the first hours of immersion and subsequent breakdown of the chitosan film, the latter resulting in localized dissolution of zinc oxide.^20^ After 15 h, corrosion products fully covering the samples were visually observed, although this observation was not documented by recording images. Hence, EIS spectra of unmodified chitosan-coated samples recorded after immersion times >15 h were not considered for data analysis. The increase in capacitance can be understood, e.g., via a loss of polymeric material on the surface. It is not possible to argue from the data gathered here whether corrosion product formation causes loss in polymer adhesion or loss of chitosan causes corrosion product formation.

Overall, the bare chitosan shows clear signs of instability. The three cross-linked systems show higher resistance values, where the constant increase with time observed in chitosan-molybdate stands out.

### 2.4 Evaluation of Resistance to Cathodic Polarization by Cyclic EIS

**Figure 7:**
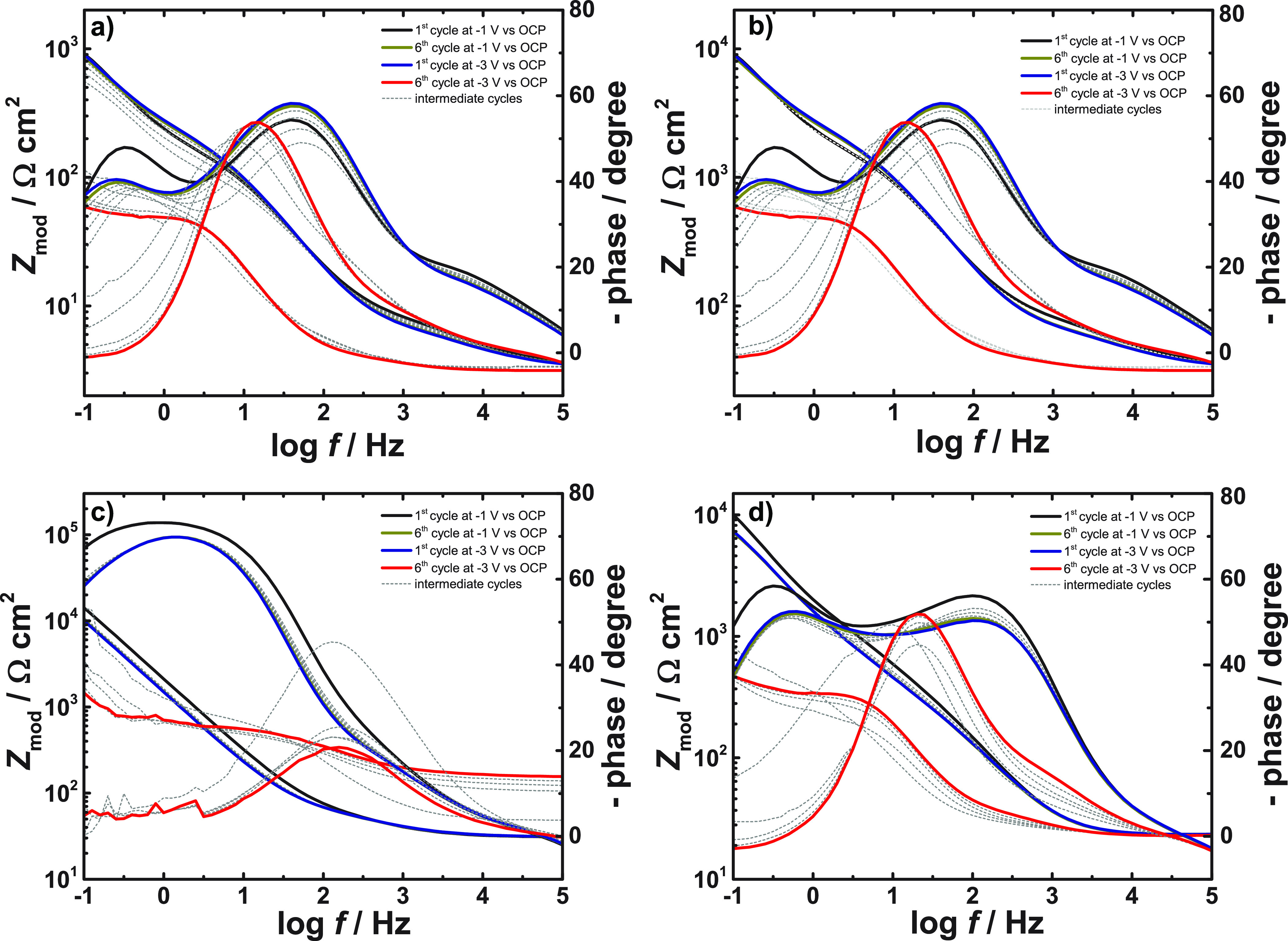
EIS spectra after different cycles of cathodic polarization recorded for (a) chitosan, (b) chitosan-silane, (c) chitosan-molybdate, and (d) chitosan-phosphate immersed in NaCl 5% at open circuit potential.

Cyclic EIS experiments were performed in order to evaluate resistance of the chitosan-based films toward cathodic polarization. Figure 7a–d show the spectra for chitosan-based films recorded by cyclic EIS. Initially all films, except chitosan-molybdate, exhibit two time constants associated most likely with water penetration (fast) and formation of passive products (slow). A distinctive feature of the evolution of the time constant is the change from exhibiting two time constants to only one time constant with a larger nonideality, i.e. a phase with a stronger deviation from −90°. Chitosan, chitosan-silane and chitosan-phosphate show a final time constant with similar magnitude and at very similar frequencies (≈ 1 Hz). However, the time constant of chitosan-molybdate shifts by 1 order of magnitude, and the corresponding negative phase moves further away from −90°.

**Figure 8:**
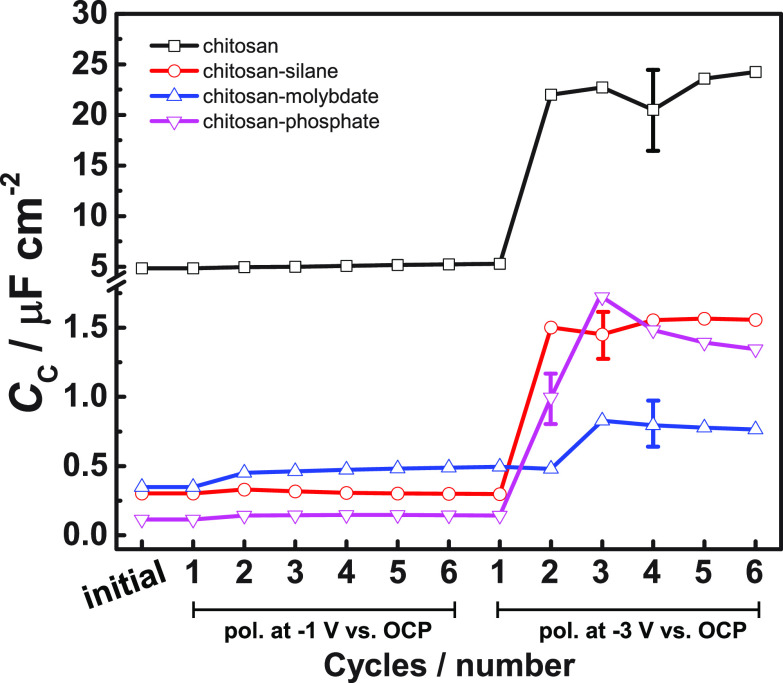
Area normalized effective characteristic capacitance for chitosan-based films calculated from the impedance modulus at the frequency of maximum time constant for the spectra in Figure 7 according to eq 3. Typical uncertainties are shown for selected points only to keep the graph legible.

Figure 8 shows a characteristic effective capacitance *C* after each cycle. This characteristic capacitance was calculated from the impedance modulus |*Z*| at the frequency *f* where the corresponding relaxation process exhibits its maximum in negative phase. The calculation starts from the definition of the impedance of a capacitor:
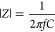
1In a system such as this, the characteristic effective capacitance must always be in series with *R*_el_. Thus, *R*_el_ must be subtracted from the measured total impedance modulus for a better estimate of the capacitance, which yields
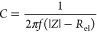
2If |*Z*| ≫ *R*_el_, *R*_el_ can conveniently be neglected. Here, for chitosan, chitosan-silane, and chitosan-phosphate, the effect of the *R*_el_, estimated from the high frequency limit, is on the order of 10% (Figure 7) and can thus be neglected. The same applies for chitosan-molybdate after the polarization at −1 V vs open circuit potential (OCP). Since we cannot accurately determine *R*_el_ for all systems here, we use the approximation
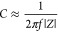
3

After the latest and most severe polarization cycles, the chitosan-molybdate film apparently exhibits the lowest capacitance. However, this low capacitance is an artifact which can be explained by neglecting the *R*_el_ in eq 2; if the appropriate correction is performed, chitosan-silane, chitosan-phosphate, and chitosan-molybdate are close to each other. (A quantitative correction for all measurements requires a stable constant value of *Z* at high frequencies, which is not obtained for all systems. Assuming that *Z* decreases as frequency increases at the highest frequencies implies that this correction is not quantitatively important for any system except chitosan-molybdate after the more severe polarization cycles. Because performing the correction quantitatively only on selected systems would result in a plot that is inconsistent, the plot contains the uncorrected data.) During the initial cathodic polarization cycles at −1 V vs OCP, the characteristic capacitance follows the trend (chitosan-molybdate) > (chitosan-silane) > (chitosan-phosphate), which is not the same as in the SKP measurements [(chitosan-molybdate) > (chitosan-phosphate) > (chitosan-silane)]. In SKP experiments, the electrolyte approaches the coated sample horizontally, forming a thin layer at the metal–polymer interface. The capacitance becomes several times higher during the second sequence, indicating that the metal is getting directly exposed to the electrolyte.

## 3 Conclusions

Chitosan-molybdate and chitosan-phosphate cross-linked films have been investigated as a conversion-like protective layer against corrosion on HDG steel. Chitosan-molybdate and chitosan-phosphate cross-linked films were prepared by ionic cross-linking of amine groups of chitosan with phosphate and molybdate anions, respectively. Such films can interact with the oxides present on HDG steel forming a layer of passive products. As shown in Figure 1, the chitosan-based film deposited on the substrate can be considered as a polyelectrolyte (multi)layer.

The short-term protection properties of the films were compared to those of an unmodified chitosan and chitosan-silane hybrid film. Both phosphate and molybdate cross-linked chitosan films formed a homogeneous layer on the metallic substrate, covering the complete substrate. Chitosan-phosphate films showed a similar resistance toward cathodic delamination as chitosan-silane films. Even though chitosan-molybdate films exhibited twice the delamination rate of chitosan-silane and chitosan-phosphate, the reaction of molybdate with the HDG surface leads to a 5-fold increase of the dominant resistance over immersion times >24 h, longer than probed in the delamination experiments. Thus, chitosan-molybdate offers a higher long-term protection against ion migration and water diffusion. All layers showed a linear time dependence of the delamination.

Measurements over a number of hours indicate that these films can potentially be used as an alternative to other methods of surface passivation such as conversion coatings as pretreatment. EIS data for both phosphate and molybdate showed a gradual increase of the dominating resistance with time, which can be associated with the release of phosphate and molybdate from the chitosan layer as result of excess of negative charges because of penetration of chloride ions. Therefore, such as system could represent a film with a reservoir of corrosion inhibitor with release triggered by ion exchange, where especially the behavior of molybdate in long-term experiments is quite promising. The corrosion inhibitors react with the HDG surface and inhibit further corrosion; the discussion of their protection mechanism is beyond the scope of this work. Thus, chitosan-phosphate and molybdate films could be applied in protection of HDG steel against corrosion specially during containment and transport at near neutral pH.

## 4 Experimental Section

### 4.1 Materials

HDG Z275 steel sheets with a metallic coating thickness of ∼20 μm, a total thickness of 1.5 mm, and a roughness *R*_a_ between 0.8 and 1.2 μm were supplied by Chemetall (Frankfurt am Main, Germany). Chitosan (weight-averaged molecular weight 650 000 g mol^–1^, min. 80% deacetylated) was obtained from Wako Pure Chemical Industries (Osaka, Japan). The epoxysilane coupling agent GLYMO (97%) was obtained from Sigma-Aldrich (Darmstadt, Germany). All chemicals were used as received unless otherwise noted.

### 4.2 Sample Preparation

HDG Z275 steel substrates (18 × 29 cm^2^) were chemically cleaned by applying ethanol and ethyl acetate, and then alkaline degreased with Gardoclean S5160 (Chemetall, Frankfurt am Main, Germany) with free alkalicity 3.9–4.1 pt. for 12 s at 60 °C with an injection pressure of 1 bar. Finally, the substrates were rinsed with water and dried with filtered pressurized air at room temperature (22 ± 2 °C). Cleaned substrates were wrapped in aluminum sheets and kept in a hermetic envelope in vacuum.

Phosphate solution (0.5 M) was prepared dissolving 8.20 g of sodium phosphate in 100 mL of deionized water and kept in glass bottles at room temperature. Molybdate solution (0.5 M) was prepared dissolving 12.1 g of sodium molybdate dihydrate in 100 mL of deionized water and kept in a glass bottle at room temperature. Chitosan solution was prepared by overnight stirring of 10 g of chitosan flakes in 1000 mL of diluted acetic acid (0.2 M) at 50 °C. The resulting solution was kept in a glass bottle at room temperature protected from light. Prior to use, an aliquot necessary for each experiment was decanted by a funnel equipped with a plastic filter of 100 μm pore size.

### 4.3 Preparation of Chitosan-Based Films

Chitosan-phosphate and chitosan-molybdate films were prepared by mixing chitosan and sodium phosphate solution or sodium molybdate solution, respectively (see section 4.2) in a volume ratio 1:1. The mixtures were stirred for several hours before application on the samples. An aliquot of ∼3 mL of the chitosan-inhibitor solution was spread over a sample and left in contact for few minutes. Subsequently, the excess was removed using a spiral bar coater of 12 μm (chitosan-molybdate) or 10 μm (chitosan-phosphate, chitosan-silane). As an extreme case in the lower end of the thickness range, for one sample, an aliquot of 1 mL of chitosan solution was dropped and spread using a spiral bar coater of 10 μm. The coated samples were left to dry at room temperature for 15 min and then thermally cured for 2 h at 100 °C. Final coated samples were stored 24 h at room temperature and protected from light prior to electrochemical experiments. This procedure yielded films of ∼3 μm in thickness for all systems, as determined by Eddy current measurements. Differences in thickness between the different systems in this work were lower than the resolution of a Eddy current thickness measurement.

As reference, for each experiment, chitosan and chitosan-silane hybrid coated samples were prepared using formulation explained elsewhere.^37^Figure 2 shows a schematic representation of the sample used for delamination studies. For delamination studies only, spreading of electrolyte over the sample surface was avoided by coating each sample with a 5% alcoholic solution of PVB to yield a top coating of ∼20 μm thickness. As control experiment, bare HDG steel samples were coated with a solution of unmodified chitosan and top-coated with PVB; delamination experiments were performed under the same conditions as described above.

### 4.4 Electrochemical Measurements

Time-dependent EIS measurements were performed continuously at ambient temperature using a multistation Gamry potentiostat in a two-electrode cell of ∼70 mL with a working electrode area of 18 cm^2^, equipped with a platinum mesh counter electrode of an electrode area 10–50× that of the working electrode. The use of the two electrode setup is inspired by DIN EN ISO 16773 and the corresponding substandards.^95^ Two electrode systems are commonly used in the industrial characterization of coatings because of technical advantages. Two electrode setups have advantages for thick coatings, though they are not preferred for freely corroding samples because of the lack of control of potential at the working electrode. For the EIS measurements, all tested samples were submerged in 5% NaCl. No additional top coating was applied for these studies. All measurements were obtained in the frequency range from 100 kHz to 100 mHz with perturbation amplitude of 10 mV. Time-dependent EIS measurements were analyzed by fitting initial data to an equivalent circuit, and by automatic fitting of the full time series to the same equivalent circuit using the Gamry analysis software.

To study the resistance of the films to cathodic polarization, a cyclic test with interleaved EIS measurements was performed. Cathodic polarization stresses organic coatings, and its use is common practice in the characterization of organic coating performance.^96−99^ In a first sequence, six cycles of cathodic polarization at −1 V with respect to initial OCP were performed for 10 min each cycle, followed by a relaxation period of 60 s, and the recording of EI spectra after each period with polarization. In a second sequence, six cycles of cathodic polarization at −3 V with respect to initial OCP were performed for 10 min each, with their respective EIS monitoring measurements. An initial EIS measurement for each sample was recorded as reference. For analyzing EIS data from this cyclic impedance test, only a simplified EIS analysis was performed. To avoid confusion between the different analysis methods, the respective details are described together with the results sections 2.3 and 2.4 of the manuscript.

Delamination experiments were performed on a commercial SKP system KM Soft Control (Wicinski - Wicinski GbR, Wuppertal, Germany) equipped with a 100 μm diameter NiCr tip in a humidity chamber with relative humidity of ∼95% at room temperature.^100^ The application of SKP to delamination studies was described at an introductory level elsewhere.^75^ Preceding each experiment, the SKP was calibrated to the standard hydrogen electrode (SHE) against Cu|CuSO_4(sat.)_.^76^ For initiation of the cathodic delamination process, an artificial defect was created at the edge and filled with 5% NaCl. Progress of the delamination front was analyzed as described elsewhere.^76^ The first point exhibiting the potential of the intact interface was taken as the position of the delamination front.

Experiments of the novel systems in this work (chitosan-molybdate, chitosan-phosphate) have been performed in triplicate, whereas experiments of the systems for which more data for similar systems are available (chitosan-silane, chitosan) were done at least in duplicate. Repeatability between samples is in a typical range for such systems, with important trends (such as the increase in *R*_2_ for molybdate, trends in delamination rates) consistently observed.
